# Total colectomy for multiple metachronous colon cancers in a patient with Lynch syndrome

**DOI:** 10.1186/s40792-015-0081-x

**Published:** 2015-09-09

**Authors:** Masatoshi Kochi, Manabu Shimomura, Takao Hinoi, Hiroaki Niitsu, Takuya Yano, Shoichiro Mukai, Hiroyuki Sawada, Masashi Miguchi, Yasufumi Saito, Tomohiro Adachi, Yasuyo Ishizaki, Hiroyuki Egi, Hideki Ohdan

**Affiliations:** Department of Gastroenterological and Transplant Surgery, Applied Life Sciences, Institute of Biomedical & Health Sciences, Hiroshima University, 1-2-3 Kasumi, Minami-ku, Hiroshima, 734-8551 Japan; Department of surgery, Hiroshima General Hospital of West Japan Railway Company, 3-1-36 Futabanosato, Higashi-ku, Hiroshima, 732-0057 Japan

**Keywords:** Lynch syndrome, *MSH2* mutation, Metachronous cancer, Total colectomy

## Abstract

Lynch syndrome (LS) is a disorder caused by mismatch repair gene mutations, which have been recognized to be associated with an increased frequency of colorectal and extracolorectal tumors. However, it remains controversial as to whether total or segmental colectomy should be performed to treat colorectal cancer in patients with LS. A 58-year-old male underwent total colectomy with ileostomy for advanced transverse colon cancer. He was also found to have LS based on his characteristic family history and the findings of a preoperative examination, including a microsatellite instability analysis of past multiple metachronous cancers. The postoperative histological findings showed mucinous adenocarcinoma without lymph node metastasis, and the loss of the *MSH2* protein expression was confirmed on an immunohistochemical examination. The present case provided important information on the clinical management of multiple developing metachronous colorectal cancers in patients with LS.

## Background

Lynch syndrome (LS) is a dominantly inherited syndrome characterized by the development of a variety of cancers in the colorectum, endometrium, skin, ovaries, urothelial tissue, small intestine, pancreas, and hepatobiliary tract [1, 2]. Lynch syndrome-associated tumors are caused by germline mutations in DNA mismatch repair (MMR) genes, including *MLH1*, *MSH2*, *MSH6*, and *PMS2* (*MLH1* and *MSH2* account for the majority of cases) [3]. Moreover, deletions of the terminal codon of the *EPCAM* gene, located upstream from the *MSH2* gene, result in silencing of the *MSH2* gene and produce a phenotype very similar to LS [4]. The loss of function of this pathway leads to microsatellite instability (MSI) with an increased risk of colorectal and extracolorectal cancer, comprising 2–7 % of all colorectal cancers (CRCs) [2, 5, 6].

The conventional identification of LS patients relies on the application of various clinical guidelines, including the Amsterdam II criteria and revised Bethesda guidelines for LS. Subsequently, a second screening, which includes an MSI analysis and/or an immunohistochemical analysis is performed for the patients who match the prior clinical guidelines. A definitive diagnosis is made according to analyses of mismatch repair genes. The clinical characteristics of this disease include a tendency to occur in the proximal colon, an increased risk of early-onset cancer, rapid progression, and a relatively good prognosis [2, 5, 7, 8]. Mutation carriers have a risk of developing CRC of approximately 50–82 % throughout their lifetime, with occasional relapses after treatment [5, 9, 10].

However, it remains controversial as to whether total or segmental colectomy should be used to treat CRC in patients with LS. Since CRC does not always develop in LS patients with a penetrance of approximately 80 % and conventional surveillance with colonoscopy may be performed as nonsurgical therapy for tumors prior to the development of advanced cancer, prophylactic total colectomy is not frequently performed in cases of Lynch syndrome, unlike that observed in familial adenomatous polyposis (FAP) [1]. Nevertheless, prophylactic total colectomy may be indicated if the frequency of CRC is high, and it is difficult to prevent progression to advanced cancer due to rapid development.

The present report describes the case of a patient with LS in whom a third colon cancer was detected in an advanced stage within 1 year after periodic colonoscopy. Total colectomy with ileostomy was administered considering the patient’s history of multiple metachronous colon and extracolorectal cancers diagnosed preoperatively according to an MSI analysis using previous cancer tissues.

## Case presentation

A 58-year-old Japanese male was referred to our hospital for the treatment of advanced transverse colon cancer diagnosed based on annual colonoscopy findings.

The patient’s past medical history included ulcerative colitis (UC), which had been treated with proctectomy and a colonic stoma in his 20s; although this condition was not currently being followed up, adenocarcinoma of the stomach was treated via distal gastrectomy at 53 years of age and primary colon cancer (of the transverse and ascending colon) treated via endoscopic mucosal resection (EMR) and segmental colectomy at 55 and 56 years of age, respectively. Additionally, colonoscopy had revealed multiple adenomatous polyps within the last 6 years.

No apparent abnormalities were observed on a physical examination except for a surgical scar on the patient’s midline and the colostomy site in the left lower quadrant of the abdomen.

The hematological laboratory data on admission were as follows: white blood cell count = 6,390/μl (normal range 4500–9000); hemoglobin = 9.6 g/dl (normal range 13.6–17.0); platelet count = 31.3 × 10^4^/μl (normal range 14.0–36.0); albumin = 4.5 g/dl (normal range 4.0–5.0); carcinoembryonic antigen = 1.8 ng/ml (normal range 0–5.0); carbohydrate antigen 19–9 = 16 U/ml (normal range 0–37.0).

**Fig. 1:**
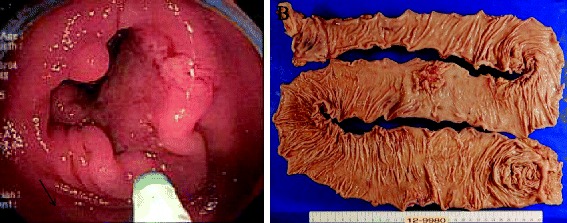
**a** Colonoscopy disclosed a type 2 tumor in the transverse colon that was histologically diagnosed as poorly differentiated adenocarcinoma. **b** Macroscopic view of the resected total colon with cancer. A type 2 tumor (50 mm) is noted

Colonoscopy disclosed a malignant appearing deep ulcerated lesion occupying half of the lumen in the transverse colon (Fig. 1a). An adenomatous polyp in the cecum was subsequently removed, without any signs of recurrence of UC. A biopsy of the transverse colon tumor showed poorly differentiated adenocarcinoma with the accumulation of mucus.

Thoracoabdominal computed tomography (CT) and positron emission tomography (PET)-CT demonstrated no systemic metastasis of colon cancer.

**Fig. 2:**
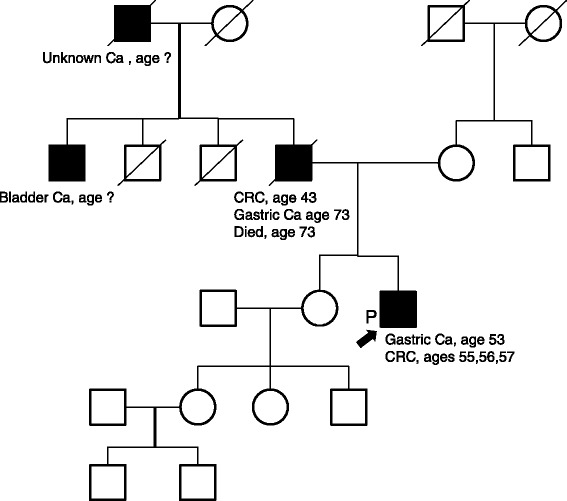
The patient’s pedigree. Three family members among two generations were affected by Lynch syndrome-associated tumors. The patient’s family satisfied the Amsterdam II criteria and revised Bethesda guidelines for a diagnosis of Lynch syndrome

**Fig. 3:**
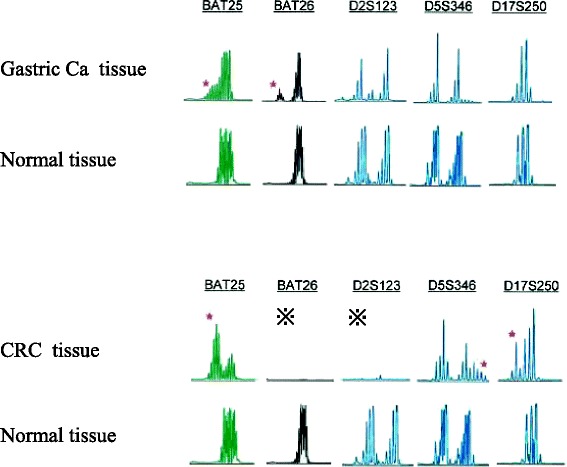
Results of the microsatellite instability (MSI) analysis. The gastric cancer and CRC tissues exhibited a high level of MSI based on the number of replication errors. ※poor in amplification

The pedigree of the patient’s family is shown in Fig. 2. His father had suffered from colon cancer at 43 years of age and gastric cancer at 73 years of age, his paternal aunt had a history of bladder/urinary tract cancer, and his paternal grandfather had died due to an unknown cancer; genetic testing was not performed in these individuals. The patient was suspected of having LS based on the Amsterdam II criteria and revised Bethesda guidelines as well as his history of metachronous cancer. Therefore, we conducted an MSI analysis of the previous gastric and colon cancer tissues to confirm the possible role of LS, the results of which revealed MSI-high status in both cancer tissues (Fig. 3). These findings strongly suggested a diagnosis of LS. Furthermore, the frequency of CRC was high, and advanced CRC was detected within one year after periodic colonoscopy. We therefore recommended that the patient undergo total colectomy.

**Fig. 4:**
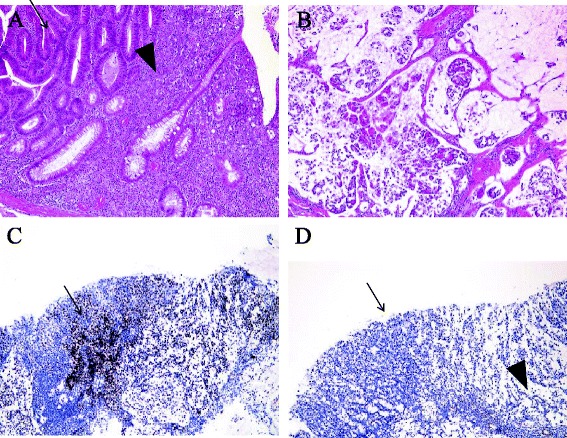
**a** Detailed microscopic view. The tumor displays heterogeneity of poorly differentiated adenocarcinoma (*arrowhead*) and well-differentiated adenocarcinoma (*arrow*). **b** The lesion consists of mucinous adenocarcinoma. **c** Immunostaining for the mismatch repair protein MLH1 clearly showed positive staining in the cancerous tissue (*arrow*) and non-cancerous tissue. **d** Immunostaining for the mismatch repair protein MSH2 showed positive staining in the non-cancerous tissue (*arrowhead*), with no staining in the cancerous tissue (*arrow*)

We successfully performed total colectomy with ileostomy after discussing the options for surgery with the patient and his family (Fig. 1b). The histopathological findings of the surgical specimen showed a type 2 tumor (50 × 40 mm) in the transverse colon exhibiting heterogeneity of poorly differentiated adenocarcinoma, mucinous adenocarcinoma, and well-differentiated adenocarcinoma. However, no lymph node metastasis was detected (T4a N0 M0 Stage IIB [according to the 7th edition of the International Union Against Cancer TNM classification, Fig. 4a, b]).

Immunostaining for mismatch repair proteins revealed clearly positive *MSH2* staining in the non-cancerous lesions, with no staining in the cancerous lesions. These findings suggested that the patient had LS related to *MSH2* deficiency (Fig. 4c, d).

The patient’s postoperative course was uneventful, and he was discharged from our hospital 29 days after undergoing surgery. Capecitabine was administered as adjuvant chemotherapy for 6 months, with no signs of recurrence for over 1 year. After genetic counseling, germline testing for MMR (*MLH1* and *MSH2*) gene revealed no mutation in *MLH1* and p.G40S (GGC > AGC) mutation in *MSH2* in heterozygote. Currently, *EPCAM* gene mutation analysis is not available in Japan.

### Discussion

We herein report a case of LS in a patient who underwent total colectomy based on his history of multiple metachronous colon and extracolorectal cancers diagnosed preoperatively according to an MSI analysis using previous cancer tissues. To confirm the diagnosis for LS, MSI status is not sufficient; germline mutation analysis which usually takes several weeks should be performed to confirm the diagnosis of LS, although it is not always available before operation. Moreover, germline mutation analysis does not always come to the conclusion if the alteration turned out to be a variant of unknown significance as we detected in *MSH2* gene as missense mutation p.G40S. Therefore, germline mutation analysis of the terminal codon of the *EPCAM* gene might be necessary to find a pathological mutation [4]. In addition, there is the report that 45 % of *MSH2* mutations are detected in multiplex ligation-dependent probe amplification (MLPA) [11]. So, MLPA might be considered for analysis of the MMR gene mutation that was suspected by MSI and immunohistochemistry (IHC) if there is no mutation detected by PCR-direct sequence.

CRC with LS develops from precursor adenomatous polyps. Several studies have suggested that this premalignant lesion related to LS tends to undergo more rapid development in LS patients than in patients who do not have LS [7, 8]. According to the pathological characteristics of LS, a high degree of lymphocyte infiltration is often observed in the cancer tissue, with the tumors being poorly differentiated and mucinous and/or comprising signet ring cell carcinoma [2, 5, 12].

Conventional surveillance with colonoscopy is recommended in LS patients, with a surveillance interval of 1 to 2 years beginning at the age of 20–25 [9, 13, 14]. The aim of this surveillance is to detect and remove adenomatous polyps before they progress to cancer [6, 10, 15, 16]. Previous studies have reported that periodic examinations with colonoscopy reduce the risk of malignancy by 62 %, with a significant reduction in mortality due to CRC [15, 17–19].

However, several reports have found that colonoscopic surveillance is associated with a small risk (approximately 6 %) of developing CRC, with 10 % of these cancers being in the advanced stage, as premalignant lesions generally develop rapidly and are morphologically flat, making them difficult to identify on colonoscopy [9, 13]. One study reported a significant rate of missed adenomas of up to 55 % using conventional colonoscopy [20].

CRC with LS is usually treated with either segmental colectomy or total colectomy with ileorectal anastomosis. The selection of the surgical procedure for CRC in LS patients is controversial due to the lack of available information regarding treatment. For example, there are no prospective studies assessing the survival benefits and QOL of large populations. Some authors recommend the use of total colectomy to treat CRC in LS patients based on findings showing that the risk of metachronous CRC following segmental colectomy ranges between 11 and 45 % over follow-up periods of 8 to 15 years [6, 9, 21].

However, total colectomy with ileorectal anastomosis is well known to be associated with a decrease in the quality of life due to an increase in stool frequency and a more liquid stool consistency [22]. Total colectomy is also considered for application as partial prophylactic therapy, as there is a possibility for the subsequent occurrence of extracolonic cancer. Additionally, since CRC does not always develop in LS patients and conventional surveillance with colonoscopy may be performed as nonsurgical therapy for tumors prior to the development of advanced cancer, prophylactic total colectomy is not often performed in cases of LS, unlike that observed in FAP [23].

Previous studies have reported that total colectomy does not significantly improve the overall survival of LS patients, and the surgical procedure should be selected based on individual patient factors and preferences [24, 25]. Therefore, total colectomy should only be offered under the following special circumstances: cases of early-onset cancer or repeated episodes of cancer development and patients in whom it is technically difficult to perform colonoscopy or those with poor compliance with surveillance examinations or who choose to undergo colectomy rather than surveillance [9]. It is important to communicate with the patient regarding the expectations and future risks associated with either option with respect to the risk of cancer in the remaining colon and the potential for functional depression after removing the entire colorectum [6, 21, 22]. The following information also assists in the selection process: the location of the tumor, existence of colonic disease, age, comorbidities, and the baseline bowel/sphincter function [24].

In the present case, despite undergoing annual colonoscopy, the patient was found to have CRC at an advanced stage. He had previously undergone proctectomy for UC, segmental colectomy for CRC and distal gastrectomy for gastric cancer. There is a possibility that the presence of abdominal adhesion due to the use of multiple surgeries made it technically difficult to adequately perform colonoscopy. Considering factors such as the patient’s age, the technical difficulty in examining the colonoscopy findings, and the frequency of metachronous CRC, we recommended that he undergo total colectomy. He had previously undergone proctectomy for UC; therefore, we chose total colectomy with ileostomy, not ileorectal anastomosis. Since the patient had undergone colostomy placement in his 20s, he was able to adapt to the ileostomy following the removal of the entire colorectum. To the best our knowledge, no previous reports have indicated a relationship between UC and LS.

Patients with LS are not often diagnosed at the time of surgery. Therefore, it is important to consider the possibility of LS in individuals with a characteristic family history, past medical history, and pathological tissue findings. If LS is suspected, the physician should attempt to perform an MSI analysis, immunohistochemistry, and germline mutation analysis. After the diagnosis, it is necessary to carefully consider the indication for total colectomy based on the patient’s history. In the present case, we were able to strongly suggest LS before surgery by conducting an MSI analysis of both previously resected specimens.

## Conclusions

There is concern regarding the selection of the surgical procedure in cases of CRC in LS patients. Prophylactic total colectomy should be considered in patients with a high frequency of CRC in whom it is difficult to prevent progression to advanced cancer due to rapid tumor development.

In addition, our findings emphasize the significance of making a preoperative diagnosis of LS. It is important to suggest LS preoperatively using MSI analyses and immunohistochemistry in cases involving a characteristic family history, past medical history and pathological findings in biopsy specimens. The present case provided important information on the clinical management of multiple developing metachronous colorectal cancers in patients with LS.

## Consent

Written informed consent was obtained from the patient for publication of this Case report and any accompanying images. A copy of the written consent is available for review by the Editor-in-Chief of this journal.

## Abbreviations

LS: lynch syndrome
MMR: mismatch repair
MSI: microsatellite instability
CRC: colorectal cancer
FAP: familial adenomatous polyposis
UC: ulcerative colitis
EMR: endoscopic mucosal resection
CT: computed tomography
PET: positron emission tomography
MLPA: multiplex ligation-dependent probe amplification
IHC: immunohistochemistry
